# Photic and Pineal Modulation of Food Anticipatory Circadian Activity Rhythms in Rodents

**DOI:** 10.1371/journal.pone.0081588

**Published:** 2013-12-04

**Authors:** Danica F. Patton, Maksim Parfyonov, Sylviane Gourmelen, Hanna Opiol, Ilya Pavlovski, Elliott G. Marchant, Etienne Challet, Ralph E. Mistlberger

**Affiliations:** 1 Department of Psychology, Simon Fraser University, Burnaby, British Columbia, Canada; 2 Institute of Cellular and Integrative Neurosciences, CNRS UPR3212 University of Strasbourg, Strasbourg, France; 3 Department of Psychology, Vancouver Island University, Nanaimo, British Columbia, Canada; Kent State University, United States of America

## Abstract

Restricted daily feeding schedules entrain circadian oscillators that generate food anticipatory activity (FAA) rhythms in nocturnal rodents. The location of food-entrainable oscillators (FEOs) necessary for FAA remains uncertain. The most common procedure for inducing circadian FAA is to limit food access to a few hours in the middle of the light period, when activity levels are normally low. Although light at night suppresses activity (negative masking) in nocturnal rodents, it does not prevent the expression of daytime FAA. Nonetheless, light could reduce the duration or magnitude of FAA. If so, then neural or genetic ablations designed to identify components of the food-entrainable circadian system could alter the expression of FAA by affecting behavioral responses to light. To assess the plausibility of light as a potential mediating variable in studies of FAA mechanisms, we quantified FAA in rats and mice alternately maintained in a standard full photoperiod (12h of light/day) and in a skeleton photoperiod (two 60 min light pulses simulating dawn and dusk). In both species, FAA was significantly and reversibly enhanced in the skeleton photoperiod compared to the full photoperiod. In a third experiment, FAA was found to be significantly attenuated in rats by pinealectomy, a procedure that has been reported to enhance some effects of light on behavioral circadian rhythms. These results indicate that procedures affecting behavioral responses to light can significantly alter the magnitude of food anticipatory rhythms in rodents.

## Introduction

Restricted daily feeding schedules in rats, mice and other species induce daily rhythms of food anticipatory activity (FAA) that exhibit formal properties of a circadian clock controlled process [1]–[3]. Food anticipatory rhythms have therefor been conceptualized as the output of food-entrainable circadian oscillators (FEOs). The location of FEOs necessary for FAA remains an open question. Circadian clock genes exhibit daily rhythms of expression in cells in many brain regions and in most peripheral organs and tissues, and in most cases, these cycles are shifted or entrained by daily feeding schedules [4]–[8]. Lesions and gene knockout studies have ruled out many of these sites of clock gene expression as necessary for food anticipatory rhythms, including the suprachiasmatic nucleus (SCN), the site of a master light-entrainable circadian pacemaker [3], [9], [10]. Some lesions and gene mutations have been found to attenuate or enhance food anticipatory activity, but a convincing case for necessity has not yet been made [9]–[11]. One interpretation of these results is that food anticipatory circadian rhythms are regulated by an anatomically distributed system involving multiple oscillators, modulating factors and entrainment pathways.

A standard protocol for inducing food anticipatory activity rhythms in nocturnal rats and mice is to limit food access to a few hours during the middle of the daily light period, when activity levels are normally low and ceiling effects of nocturnal activity are avoided. Activity during the light period is thought to be suppressed by sleep-promoting outputs from the SCN pacemaker [12] and by a direct effect of light (so-called ‘negative masking’ of activity) [13]–[16]. Despite these impediments to spontaneous daytime activity, FAA to daytime meals is normally substantial. This may be due in part to an active mechanism for inhibiting SCN output during the day that is recruited by restricted feeding schedules [17], [18]. Whether masking effects of light are also centrally inhibited during restricted feeding is unknown, and light has largely been overlooked as a potential modulator of food anticipatory rhythms. Masking effects of light are mediated by intrinsically photoreceptive, melanopsin-containing retinal ganglion cells [19], [20]. Although the central pathways that mediate the direct effects of light on activity and other brain functions have not been fully delineated, masking effects of light can be altered by lesions [17], [18] and gene knockouts (e.g., dopamine D2 receptors) [21]. It is therefor conceivable that effects of some lesions or knockouts on FAA are mediated in whole or in part by alterations in behavioral responses to light.

To evaluate the plausibility of light as a confound variable in neurobiological analyses of food-entrained activity rhythms, we compared daytime FAA in rats and mice housed in standard full photoperiods (continuous light exposure for 12h/day) with FAA in rats and mice maintained in a skeleton photoperiod consisting of two daily 60 min pulses of light simulating dawn and dusk. Skeleton photoperiods presumably correspond more closely to natural patterns of light exposure in nocturnal, semifossorial rodents, and are sufficient to stably entrain circadian rhythms in laboratory rats and mice [22]–[24]. We then quantified FAA in rats subjected to pinealectomy (PnX). The pineal gland is among the few peripheral tissues in which circadian processes are not reset by restricted feeding schedules when the SCN pacemaker is entrained to light [25]–[30]. However, PnX is thought to amplify some behavioral and circadian responses to light [31]–[34]. If light normally opposes the expression of FAA to a daytime meal, then FAA may be enhanced in rats and mice entrained to a skeleton photoperiod, and may be attenuated in rats lacking a pineal gland. We observed significant effects of each of these manipulations in the predicted direction. Together, the results indicate that daytime FAA in nocturnal rodents can be altered by procedures that affect light.

## Methods

### Ethics statement

The research described in this manuscript was approved by the institutional animal research ethics boards at Simon Fraser University and the University of Strasbourg.

### Experiment 1. Skeleton photoperiod: rats

**Animals, surgery and apparatus.** Young, adult male Sprague Dawley rats (N = 15, 225–250 g, Charles River PQ) were housed for 2 weeks in groups of 4–5 in plastic cages with litter, under a 12:12 light-dark (LD) cycle. The rats were then anesthetized with ketamine (90 mg/kg), zylazine (9 mg/kg) and isoflurane (0.5%–2.0%, as needed) and implanted (intraperitoneal, via laparotomy) with radiofrequency transponders (ER-4000, Mini-Mitter, Inc., Sunriver, Oregon). The rats were then housed singly in standard clear plastic cages (45×24×20 cm) with litter. Each cage was placed on an ER-4000 receiver platform inside an individual sound attenuating isolation chamber in a climate-controlled vivarium. The receiver was monitored continuously using the Vital View data acquisition interface and software (Mini-Mitter, Inc).

**Lighting and feeding schedules.** Rats were assigned to full photoperiod (FPP, N = 7) or skeleton photoperiod (SPP, N = 8) groups. The full photoperiod consisted of 12h light (∼70 lux) and 12h dark. The skeleton photoperiod consisted of 1h light (∼70 lux), 10h dark, 1h light and 12 h dark. The 1h light pulses simulate dawn and dusk light exposure, and stably entrain circadian rhythms in nocturnal rodents [22]–[24]. The rats were maintained under these photoperiods for 23 days with food (Purina rat chow #5001) and water available ad-libitum. The rats were then food deprived overnight, and provided food for 3h each day beginning 6h after lights-on (Zeitgeber Time 6, where ZT12 is lights-off by convention). On day 9 of restricted feeding the scheduled meal was omitted, to determine how any masking effects of light on the amount of food anticipatory activity are affected by increased hunger. After day 25 of restricted feeding, ad-libitum food access was restored, and the rats were maintained in constant dark (DD) for 13 days, to assess whether any group differences in FAA might be related to a difference in the phase of entrainment to LD. The rats were then re-entrained to a full photoperiod for 1 week. The rats previously entrained to the full photoperiod were then switched to a skeleton photoperiod (lighting treatment groups reversed) for 1 week. Food was then removed overnight and the restricted feeding schedule was reinstated for 18 days. To further assess acute effects of light and dark on FAA, on day 15, from ZT0-6, the rats in the skeleton photoperiod group were exposed to light while the rats in the full photoperiod group were exposed to dark. On day 17, at ZT4.5, the rats in the skeleton photoperiod group were exposed to a 90 min light pulse, while the rats in the full photoperiod group were exposed to 90 min dark pulse.

### Experiment 2. Skeleton photoperiod: mice

Adult male C57BL6J mice (N = 5, Janvier labs, Saint Berthevin, France) were housed in individual cages equipped with a wheel (10 cm diameter) under a 12:12 LD cycle (∼150 lux during the light period). The mice were restricted to a 6-h daily meal (ZT6-12) for 2 weeks in the full photoperiod, 2 weeks in a skeleton photoperiod and 2 final weeks in the full photoperiod. The skeleton photoperiod consisted of 1h light (∼150 lux), 10h dark, 1h light and 12 h dark. Access to food (mouse chow SAFE105, SAFE, Augy, France) was controlled automatically by the Fasting Plan system (Intellibio, Seichamps, France). Water was available ad-libitum. Wheel revolutions were recorded every 5 min (Circadian Activity Motor System, INSERM, France).

### Experiment 3: Pinealectomy: rats

Young, adult male Sprague Dawley rats (N = 17, 225–250 g, Charles River PQ) were anesthetized with ketamine (90 mg/kg), zylazine (9 mg/kg) and isoflurane (0.5%–2.0%, as needed) and placed in a stereotaxic frame for surgical removal of the pineal gland (N = 9) or sham surgery (N = 8). A hole was drilled in the skull, the superior sagittal sinus was carefully deflected to minimize bleeding, and the pineal gland was removed using fine tweezers with the aid of a dissecting scope. Pinealectomies (PnX) were confirmed at the end of the study by melatonin assay (Yerkes National Primate Research Center, Emory University) on serum samples collected early in the dark period. After surgery the rats were housed in individual plastic cages in isolation cabinets. The rats were first tested for masking effects of light, and were then tested for anticipatory activity to a scheduled daily meal. Masking was assessed in two ways. All rats were maintained in LD 12:12 (∼70 lux) with ad-lib access to food. Four rats in each group had access to a running wheel. After 7 days of baseline recording, the LD cycle was changed to LD 2:2 for two days, followed by one day of DD. LD 2:2 consisted of 2h of light alternating with 2h of dark, starting at lights-on. Running wheels were then removed, and all rats were re-entrained to LD 12:12 for 14 days, with locomotor activity recorded using passive infrared motion sensors positioned above the cage. The 3-day sequence of LD 2:2 (2 days) followed by DD was repeated. The rats were then maintained in LD 12:12 with food ad-libitum for 4 weeks, after which food was restricted to a 3h daily meal, starting with an overnight food deprivation. After 22 days, the rats were food deprived for 68h in constant dark. The ZT6-9 restricted feeding schedule was then resumed for 2 days. To determine whether differences in FAA between PnX and sham lesion animals were related to the time of day of feedings, the mealtime was shifted to ZT9-12 for 22 days and to ZT3-6 for 20 days. A second food deprivation test in DD was conducted at the end of the ZT3-6 feeding schedule.

### Data analysis

For rat and mouse studies, activity data were summed in 1 or 5 min bins using the Clocklab data acquisition interface and software (Actimectrics, Evanston, IL, USA) or the Circadian Activity Motor System software, respectively. Data were then averaged in 10 min bins and visualized as actograms and average waveforms using Clocklab. The daily distribution of activity during ad-lib food access was quantified by a nocturnality ratio (percent of total daily activity occurring during lights-off). FAA was quantified by summing activity counts during the 2h preceding mealtime and expressing these as a ratio relative to total activity occurring from ZT12-24 (corresponding to the dark period in a full photoperiod, and to the biological night in a skeleton photoperiod). The period (τ) of the circadian activity rhythm free-running during the last 10 days of DD was quantified by a regression line fit to acrophases of a cosine function fit to the daily activity data. Extrapolation of the regression line back to the last day of LD provided a measure of the phase of entrainment to LD (φLD). Group differences in daily activity levels, nocturnality, FAA ratios, FAA counts, τ and φLD were evaluated by within and between group ANOVAs and t-tests as appropriate. Data are presented as means ± SEM.

## Results

### Experiment 1. Skeleton photoperiod: rats

**Figure 1:**
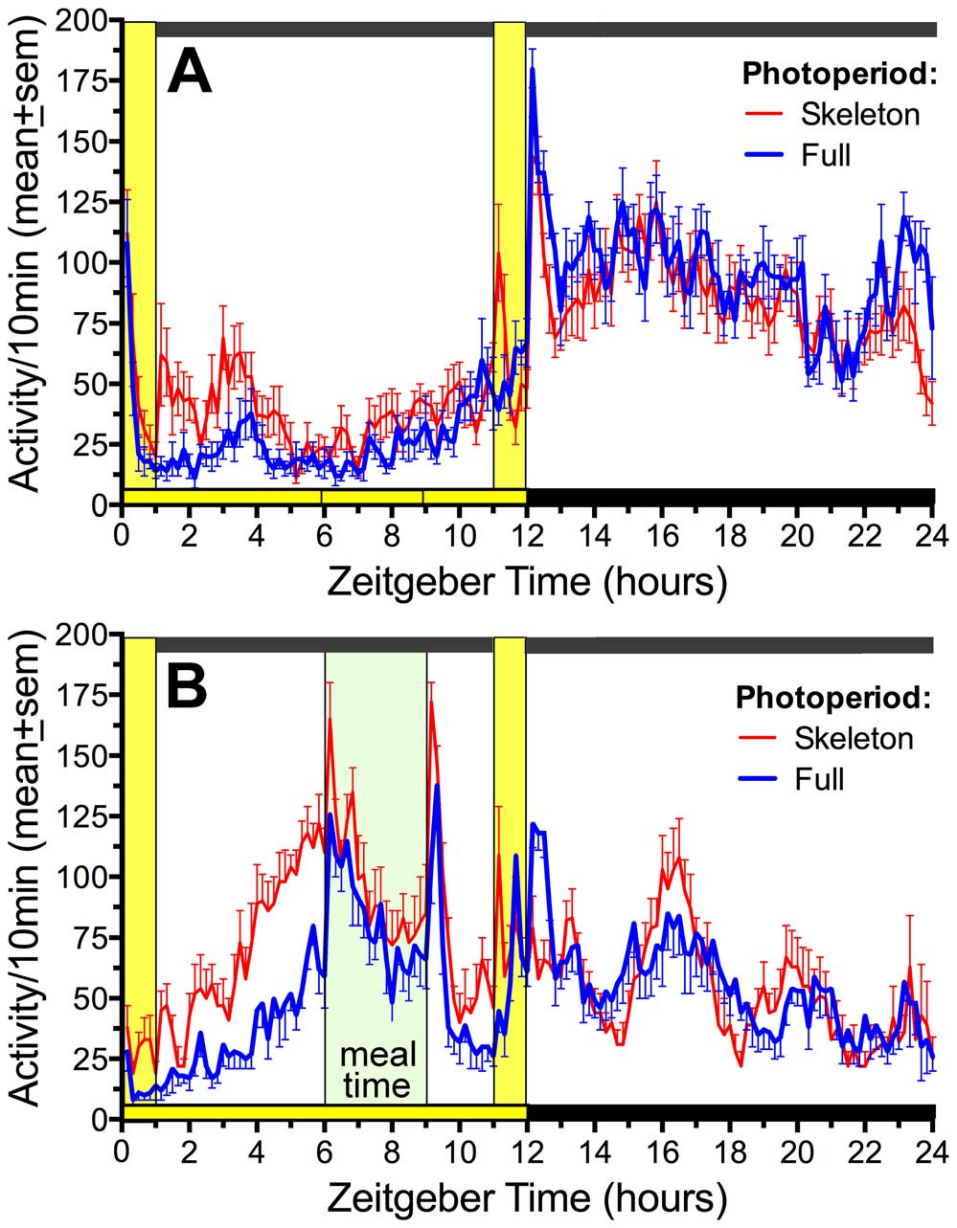
Group mean (± sem) waveforms of activity measured by telemetry in rats maintained in skeleton (red curve) and full photoperiods (blue curve). A. Food available ad-libitum. B. Food available for 3-h/day (green shading). The LD cycle during the full photoperiod is indicated by the horizontal yellow and black bars above the abscissa. Light exposure in the skeleton photoperiod is indicated by the vertical yellow bars. Time is plotted in 10 min bins, from the beginning of lights-on (Zeitgeber Time 0).

During ad-lib food access prior to daytime food restriction all rats were stably entrained to LD. There was no group difference in total daily activity (counts/day, SPP  =  8989±320, FPP = 8843±382, *p* = .78), but the skeleton photoperiod group exhibited a significantly lower nocturnality ratio (.68±.01 Vs.77±.01, *t*_(14)_  =  6.33, p<.0001; Fig. 1a) due to increased daytime (ZT0-12) activity (p<.01) and a trend for less nighttime (ZT12-24) activity (p = .07).

**Figure 2:**
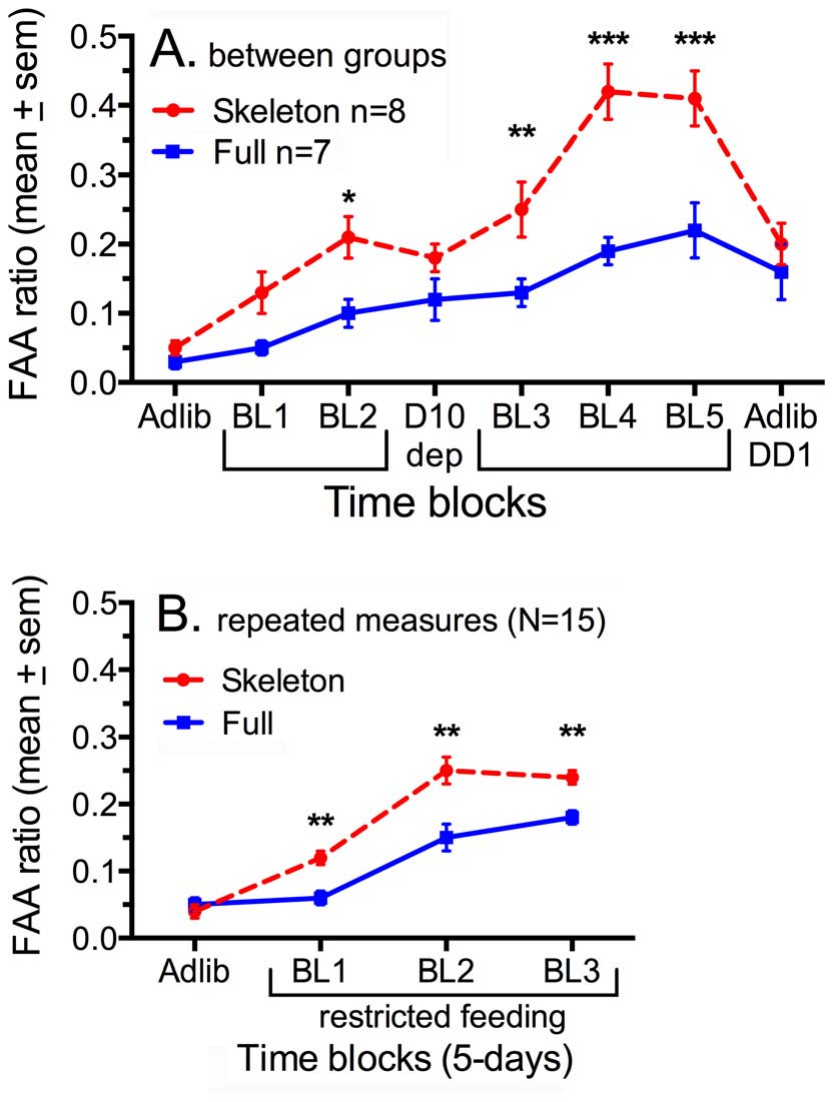
Group mean (± sem) food anticipatory activity (FAA) ratios of rats in a skeleton photoperiod (red dashed curve) and a full photoperiod (blue curve). Ratios were calculated by dividing the activity counts registered during the 2h prior to mealtime (ZT4-6) by total activity occurring from ZT12-24. A. FAA ratios for skeleton and full photoperiod groups averaged in 5-day blocks during ad-lib food access and 25 days of restricted food access (BL1-5). No food was provided on day 9 of restricted feeding, and thus day D10 is plotted separately. B. FAA ratios for the first 3 blocks of 5 days of restricted feeding, for all 15 rats tested under both the skeleton and the full photoperiods cycles (within subject, counterbalanced for order). Differences between groups: *p<.05, **p<.01, ***p<.001.

When food was restricted to ZT6-9 for 25 days, total daily activity was modestly increased (5±3%) in the skeleton photoperiod group and decreased (-9±4%) in the full photoperiod group (p = .014). The difference was almost entirely due to markedly increased FAA in the skeleton photoperiod group by comparison with the full photoperiod group (Fig. 1b). FAA ratios, averaged in blocks of 5 days (excluding days 9 and 10), were significantly higher in the skeleton photoperiod group by the second block of restricted feeding (F_(1,104)_  =  51.6, p<.0001; Fig. 2a). Food intake during the 3-h daily meals gradually increased during the first week in both groups, with no group differences across the 25 day schedule (mean intake  =  15.7±1.2 vs 15.7±1.5 g/day; Fig. 2c).

On day 9 of restricted feeding, the meal was omitted. On the following day, the food anticipation ratios did not differ between groups (Fig. 2a), indicating that the effect of photoperiod on FAA is reduced when the deprivation state is increased.

After day 25 of food restriction, ad-lib food access was restored and the rats were maintained in DD for 2 weeks. All of the rats exhibited a free-running activity rhythm with a τ>24h. There were no significant group differences in either τ (SPP =  24.29±.04, FPP =  24.29±.06, *t*_(14)_  =  1.07, p = .3) or φLD (SPP =  21.47±.54, FPP =  20.85±.40, *t*_(14)_  =  0.88, p = .39), inferred by extrapolation of a regression line through daily acrophases. Group differences in FAA were therefor not due to group difference in the phase of the LD entrained pacemaker.

After re-entrainment to skeleton and full photoperiods, with the groups reversed, a second round of restricted feeding was initiated. Over the first 15 days, FAA ratios were again greater in the skeleton photoperiod group (FPP  = .13±.01 Vs. SPP  = .21±.01, *t*_(14)_  =  6.49, p<.0001). Data from the first and second rounds of food restriction were combined, yielding a within-subjects (N = 15) comparison of FAA on the two photoperiods for the first three blocks of 5 days. FAA ratios were greater in the skeleton photoperiod condition in each of these time blocks (F_(1, 104)_  = 51.6, p<.0001; Fig. 2b).

**Figure 3:**
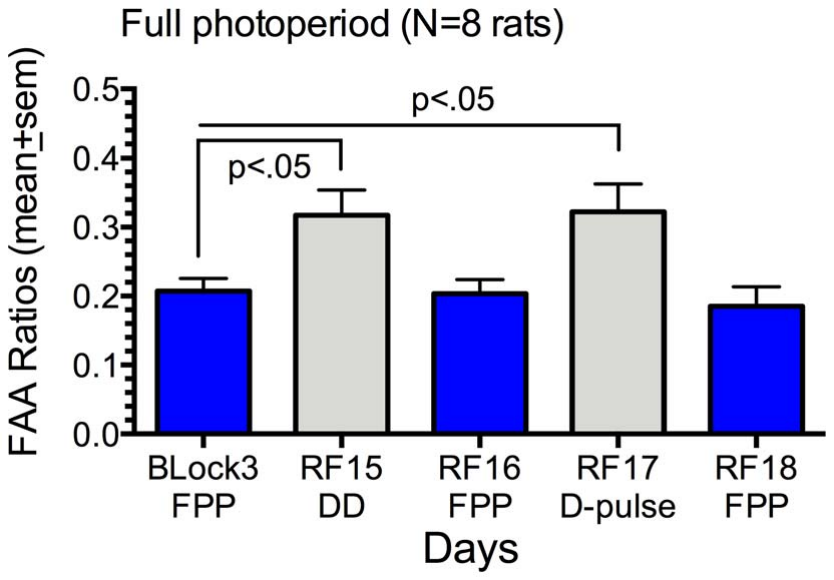
Effects of dark exposure on food anticipation ratios of rats entrained to a full photoperiod (FPP). Ratios are plotted for restricted feeding days 10–14 (block 3), day 15 (DD, no light from ZT0-6 prior to mealtime), day 16 (normal full photoperiod), day 17 (D-pulse, lights off for 90 min prior to mealtime) and day 18 (full photoperiod).

On day 15 of the second round of restricted feeding, rats in the skeleton photoperiod group were exposed to light continuously from ZT0 to ZT6 (mealtime), while rats in the full photoperiod group were exposed to dark. On day 17 rats in the skeleton photoperiod were exposed to light from ZT4.5-ZT6, while rats in the full photoperiod were exposed to dark. In the full photoperiod group, turning the lights off significantly increased the FAA ratio on both day 15 and day 17, by contrast with the previous 5 day block (F_(4,35)_  = 4.96, p = .0028; Fig. 3). FAA counts on these days changed in the same direction as the ratios, but the differences did not reach statistical significance (F_(4,28)_  = 1.78, p = .16). In the skeleton photoperiod group, turning the lights on did not significantly alter FAA ratios (F_(4,24)_  = 0.78, p = .55), although premeal (ZT4-6) activity counts showed a trend to be lower on both light exposure days (F_(4,24)_  =  2.54, p = .065).

### Experiment 2. Skeleton photoperiod: mice

**Figure 4:**
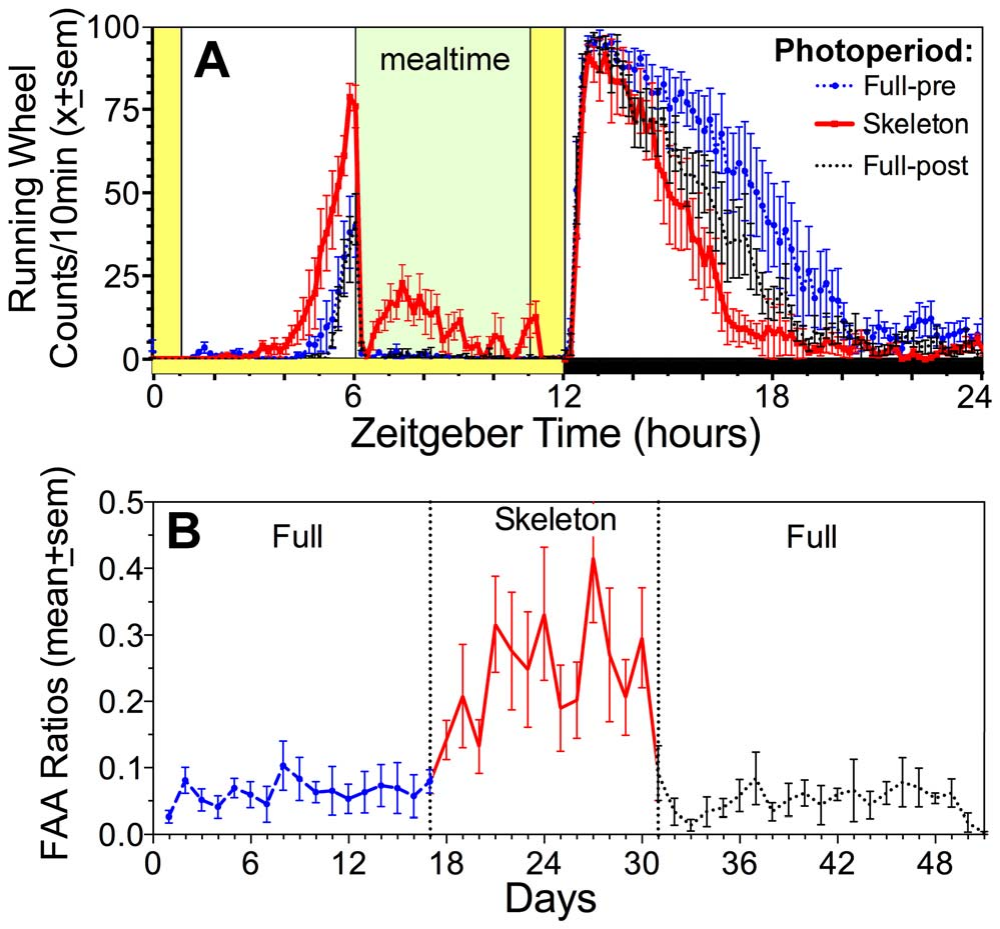
Food anticipatory activity in mice recording under a full photoperiod and skeleton photoperiods. A. Group mean (± sem) waveforms of wheel running activity in food restricted mice entrained, to a full photoperiod (blue dashed curve), a skeleton photoperiod (red solid curve) and again a full photoperiod (black dashed curve), in successive two week blocks. The waveforms were obtained by averaging the second week on each LD schedule. The 6-h daily mealtime is denoted by green shading. The LD cycle during the full photoperiod is indicated by the horizontal yellow and black bars along the horizontal axis. Light exposure during the skeleton photoperiod is indicated by the vertical yellow bars. Time is plotted in 10 min bins, from the beginning of the lights on (Zeitgeber time 0). B. Group mean (± sem) food anticipatory activity ratios calculated for each day of food restriction, by dividing activity during the 2h prior to mealtime (ZT4-6) by activity from ZT12-24.

FAA in wheel counts summed from ZT4 to ZT6 prior to mealtime was significantly increased in the skeleton photoperiod compared to both the preceding and the following full photoperiods (FPP1: 1069±367, SPP: 2921±691, and FPP2: 1006±396 wheel revolutions; F_(2,8)_  = 7.2, p = .017; Fig. 4a). Both the duration and the peak of FAA were higher in the skeleton photoperiod condition. FAA ratios were also much higher in the skeleton photoperiod compared to FPP1 and FPP2 (18.6±3.3 vs. 5.4±2.2 and 5.8±1.8%, respectively; F_(2,8)_  = 9.7, p<.01; Fig. 4b). Food intake was not significantly changed in the skeleton photoperiod group (3.2±0.1 g) by comparison to the first (3.3±0.1 g) and the second full photoperiods (3.2±0.2 g; F_(2,8)_  = 1.8, p = .22). By contrast, body weight during restricted feeding was significantly changed according to the successive photoperiodic conditions (FPP1: 21.3±0.6, SPP: 22.0±0.4, and FPP2: 22.3±0.5 g; F_(2,8)_  = 9.4, p = .008). Because the increase in body weight during the skeleton photoperiod was not reversed in subsequent full photoperiod, but instead was enhanced, this gain in body weight is likely due to the effect of time.

### Experiment 3. Pinealectomy: rats

**Figure 5:**
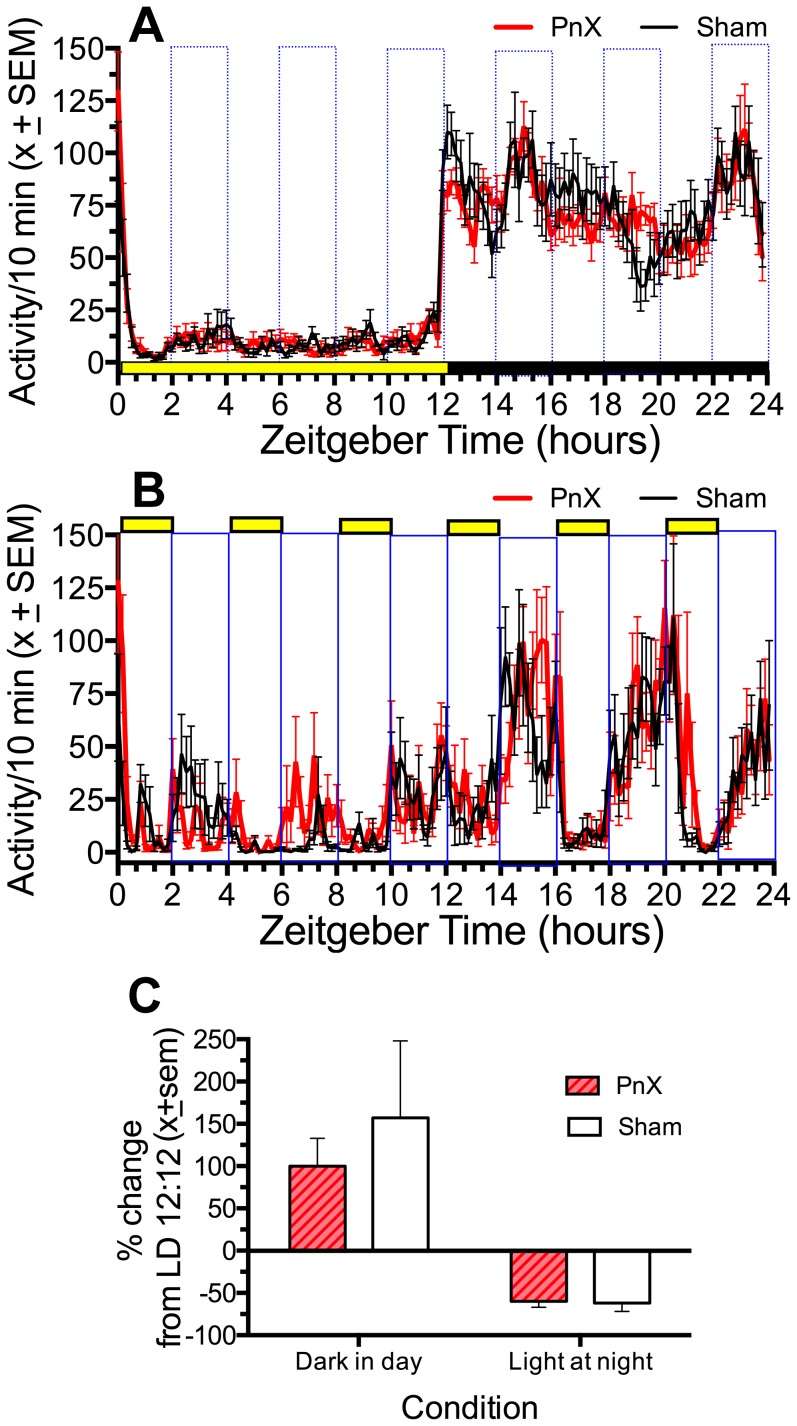
Group mean (± sem) waveforms of activity measured by motion sensors in pinealectomized (PnX, red curves) and sham lesion (black curves) rats with food available ad-libitum. A. LD 12:12, 5 days averaged. B. LD 2:2, 2 days averaged. Lights-on is indicated by horizontal yellow bars. C. Percent change of activity occurring on the LD 2:2 days compared to the preceding block of LD 12:12 days, when the lights were off during the ‘day’ (sum of ZT2-4, 6-8, 10-12) and on at night (ZT12-14, 16-18, 22-24). The group differences were not statistically significant.

**LD masking tests.** Contrary to predictions, PnX and Sham rats showed equivalent direct effects of light and dark on activity. In a preliminary test using running wheels in LD 12:12, nocturnality ratios (percent of total daily activity occurring at night) for wheel running were nearly 100% in both groups (PnX  =  97±2%, Sham rats  =  99±1%). To avoid ceiling effects, running wheels were not used further. Nocturnality of activity measured by motion sensors was lower but again did not differ by group, either in LD 12:12 (PnX =  93± 1%, shams  =  90±1%, p = .30) or during one day of constant dark (PnX =  72±2%, shame  =  71±3%). During 2 days of LD 2:2, activity was increased (positive masking) when the lights were off during the ‘day’ (ZT0-12) and was decreased (negative masking) when the lights were on at ‘night’ (ZT12-24), but the percent change from the corresponding hours in LD 12:12 did not differ between groups (F_(1,28)_  = .38, p = .5; Fig. 5)

**Figure 6:**
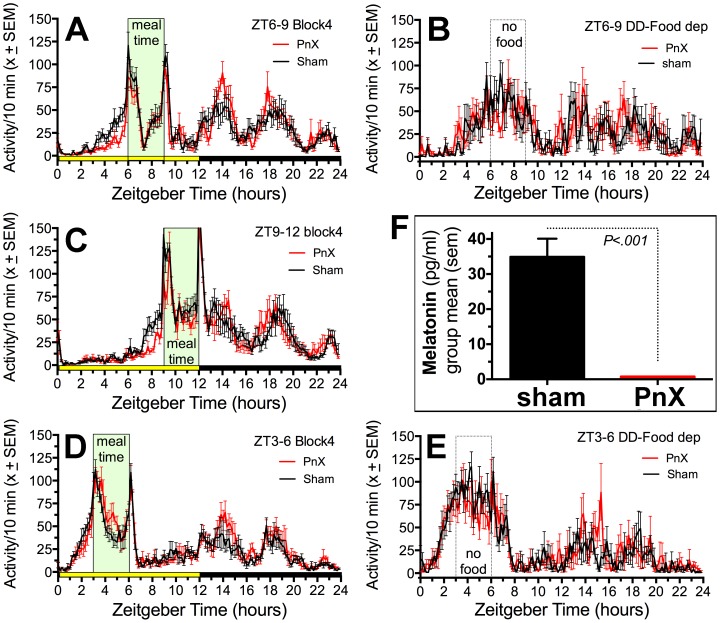
Group mean (± sem) waveforms of activity measured by motion sensors in pinealectomized (PnX) and sham rats during scheduled feeding. A. ZT6-9 mealtime; B. Food deprivation day in constant dark after last ZT6-9 feeding; C. ZT9-12 mealtime, D. ZT3-6 mealtime, E. Food deprivation day in constant dark after last ZT3-6 feeding; F. Group mean (± sem) melatonin levels in sham (solid black bar) and PnX (red line) rats from serum samples collected 2–4 hours after lights-off, when pineal melatonin secretion is normally high. In Panels A, C and D, mealtime is highlighted in green and the LD cycle is indicated by yellow and black bars along the horizontal axis.

**Figure 7:**
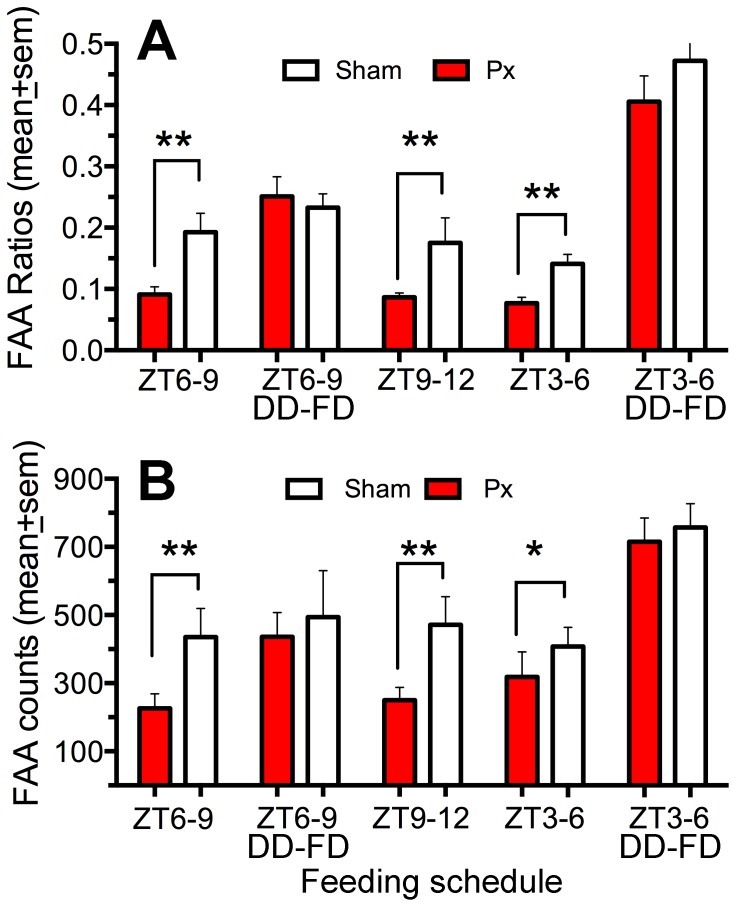
Food anticipatory activity (FAA) of pinealectomized (Px) rats (red bars) and sham rats (white bars). A. FAA ratios. B. FAA counts. Data are group means (± SEM) during the last 5 days of restricted feeding at ZT6-9, 9-12 and 3-6 (where lights-on  =  ZT0), and during one day of total food deprivation in constant dark (DD-FD) after the ZT6-9 and ZT3-6 schedules. Differences between groups: *p<.05, **p<.01.

**Food anticipatory activity.** Despite the absence of predicted group differences in masking responses to light, during restricted daytime feeding there was a significant effect of group on FAA counts (F _(1,15)_  = 4.68, p<.05) and FAA ratios (F_(1,15)_  = 14.40, p = .0018). Food was first provided from ZT6-9, and then shifted to ZT9-12 and ZT3-6 at 3 week intervals. Both groups exhibited robust FAA, beginning 2-3 h before each of mealtime (Fig. 6). Total daily activity did not differ, but the sham rats exhibited more activity during the 2-h prior to each mealtime and a significantly higher anticipation ratio (Fig. 7a,b). When the lights were turned off for one day, the group differences in food anticipation counts and ratios in the ZT6-9 and ZT3-6 conditions were absent. Notably, with the lights off prior to mealtime, FAA counts and ratios increased in both groups, but more so in the PnX group (Fig. 7a,b).

**Melatonin assay:** Successful removal of the pineal glands was confirmed by melatonin assay on serum samples collected early in the dark period, when melatonin levels normally rise. There was a marked difference between groups, with all of the PnX rats exhibiting very low levels (< 1.5 pg/ml; Fig. 6f).

## Discussion

The results of these experiments demonstrate that food anticipatory activity can be modulated by a direct effect of light. In Experiment 1, rats entrained to a skeleton photoperiod, with no light during the middle of the day when food was available, showed markedly enhanced FAA by comparison with rats exposed to daytime light in a full photoperiod. This effect was reversible, and premeal activity in rats entrained to a full photoperiod could be acutely increased by turning the lights off. The skeleton photoperiod did not alter the phase of entrainment of nocturnal activity, or free-running τ in subsequent DD, indicating that mealtimes under the two lighting conditions were occurring at the same phase of the LD-entrained pacemaker. In Experiment 2, conducted independently using mice, food anticipatory wheel running was again reversibly enhanced by exposure to a skeleton photoperiod.

In Experiment 3, the PnX rat was used as a potential model for a lesion-induced increase in negative masking by light. Both positive and negative masking were clearly evident in LD 2:2 but there was no effect of PnX on these responses. It is possible that group differences might have emerged if dimmer light had been used. Nonetheless, despite the lack of evidence for an effect of PnX on behavioral responses to light during ad-lib food access, the PnX rats did exhibit a significant reduction of FAA by comparison with the sham rats. This group difference was evident at all three mealtimes tested, and was absent when the lights were turned off for one day on two occasions. Importantly, pre-meal activity counts and anticipation ratios increased in both groups when the lights were off, and this increase was greater in the PnX rats, suggesting a greater suppression of FAA by light in this group. The pineal gland is one of the few circadian tissues that does not entrain to feeding schedules independently of the SCN [25]–[29], although it is food-entrainable in SCN-ablated rats [30]. The fact that removal of this structure is associated with a decrease in FAA when the lights are on, but not when the lights are off, underscores the importance of evaluating food anticipation under different lighting conditions or phases of the SCN pacemaker.

The effects of lighting conditions on food anticipatory activity observed in the present experiments raise the possibility that alterations in FAA following neural or genetic manipulations could in some cases be secondary to altered behavioral responses to light and dark. An example from our own prior work is the effect of neonatal monosodium glutamate (MSG) on FAA in rats [35]. MSG decreases the number of neuropeptide Y-containing neurons in the arcuate nucleus and significantly increases the amount of FAA to a daytime meal. One interpretation of these results is that the lesion blocks an inhibitory effect of the appetite-suppressing adipocyte hormone leptin on the expression of food seeking activity. However, MSG treatment also eliminated the inhibitory effect of light on locomotor activity, assessed using a 2:2 LD cycle. Therefore, an alternative, and testable, interpretation is that FAA was enhanced by a reduced activity-suppressing effect of light. This alternative interpretation may not be correct, given that FAA has also been reported to be enhanced in *ob:ob* mice deficient in leptin [36], but it does illustrate the need for evaluation of lighting effects to interpret FAA phenotypes.

The mechanisms by which lighting schedules affect FAA remain to be clarified. Increased FAA under skeleton photoperiods in the present experiments was not due to smaller meals or larger body weight loss that would enhance the metabolic impact of restricted daytime feeding and thereby potentiate FAA. The alterations in FAA can thus be attributed to altered behavioral responses to light. Light promotes sleep in nocturnal rodents [15], possibly via direct or indirect retinohypothalamic activation of ventrolateral preoptic sleep-inducing neurons; the absence of photic input to sleep promoting circuits during the mid-day in the skeleton photoperiod may thus favor wakefulness. Alternatively, darkness during the mid-day may stimulate arousal systems in the brain (e.g., lateral hypothalamic orexin neurons) [37]. Both possibilities may jointly promote the intensity of FAA under skeleton photoperiodic conditions. The mechanism by which PnX decreases FAA poses an interesting question. Pineal melatonin secretion is limited to the biological night and should be at low levels in the middle of the light period, when FAA to a meal at ZT6 occurs. Why removing a hormone present only at night should affect premeal activity in the middle of the light period is unclear. PnX has been shown to disrupt expression of circadian clock genes in the dorsal striatum [38], and other work suggests that the dorsal striatum plays a role in the expression of FAA (Steele et al, in preparation). How the presence of light would gate an effect of PnX on food-entrainable circadian processes in the striatum remains to be determined. In conclusion, environmental lighting can be a potent modulatory factor in the expression of FAA induced by restricted feeding.
